# The Performance and Evolutionary Mechanism of *Ganoderma lucidum* in Enhancing Selenite Tolerance and Bioaccumulation

**DOI:** 10.3390/jof10060415

**Published:** 2024-06-08

**Authors:** Mengmeng Xu, Qi Meng, Song Zhu, Ruipeng Yu, Lei Chen, Guiyang Shi, Ka-Hing Wong, Daming Fan, Zhongyang Ding

**Affiliations:** 1 School of Food Science and Technology, Jiangnan University, Wuxi 214122, China; xmm900801@163.com (M.X.); fandm@jiangnan.edu.cn (D.F.); 2 National Engineering Research Center for Cereal Fermentation and Food Biomanufacturing, Jiangnan University, Wuxi 214122, China; mq15006177263@163.com (Q.M.); leichen@jiangnan.edu.cn (L.C.); gyshi@jiangnan.edu.cn (G.S.); 3 Jiangsu Provincial Research Center for Bioactive Product Processing Technology, Jiangnan University, Wuxi 214122, China; 4 State Key Laboratory of Food Science and Resources, Jiangnan University, Wuxi 214122, China; zhusong@jiangnan.edu.cn (S.Z.); yuruipeng@jiangnan.edu.cn (R.Y.); 5 Research Institute for Future Food, Department of Food Science and Nutrition, The Hong Kong Polytechnic University, Hong Kong 999077, China; kahing.wong@polyu.edu.hk

**Keywords:** adaptive evolution, bioaccumulation, biotransformation, physiological phenotype, transcriptomics

## Abstract

Background: Selenium (Se) pollution poses serious threats to terrestrial ecosystems. Mushrooms are important sources of Se with the potential for bioremediation. Pre-eminent Se resources must possess the ability to tolerate high levels of Se. To obtain Se-accumulating fungi, we isolated selenite-tolerance-enhanced *Ganoderma lucidum* JNUSE-200 through adaptive evolution. Methods: The molecular mechanism responsible for selenite tolerance and accumulation was explored in *G. lucidum* JNUSE-200 by comparing it with the original strain, *G. lucidum* CGMCC 5.26, using a combination of physiological and transcriptomic approaches. Results: *G. lucidum* JNUSE-200 demonstrated tolerance to 200 mg/kg selenite in liquid culture and exhibited normal growth, whereas *G. lucidum* CGMCC 5.26 experienced reduced growth, red coloration, and an unpleasant odor as a result of exposure to selenite at the same concentration. In this study, *G. lucidum* JNUSE-200 developed a triple defense mechanism against high-level selenite toxicity, and the key genes responsible for improved selenite tolerance were identified. Conclusions: The present study offers novel insights into the molecular responses of fungi towards selenite, providing theoretical guidance for the breeding and cultivation of Se-accumulating varieties. Moreover, it significantly enhances the capacity of the bio-manufacturing industry and contributes to the development of beneficial applications in environmental biotechnology through fungal selenite transformation bioprocesses.

## 1. Introduction

Selenium (Se) is a naturally occurring abundant element in the environment, but becomes a pollutant at high levels, particularly in the form of oxyanions selenate and selenite. The cycling of Se between the ocean, terrestrial environment, and atmosphere in the environment is primarily mediated by intricate chemical and biological transformations [1,2], and both natural processes and human activities contribute to the release of Se into the atmosphere. Exposure to unnaturally high concentrations of Se can have lethal effects on humans and easily enters the food chain through plants and organisms. The consumption of more than 400 μg of Se per day can result in adverse effects such as hair loss, liver necrosis, onychorrhexis, cerebral edema, hepatic injury, and neurotoxicity [1]. Bioremediation, which relies on biological processes to degrade, transform, decompose or remove Se, is considered an alternative to conventional physicochemical methods due to its low economic cost and environmental impact, exhibiting the advantages of non-secondary contamination, economic benefits, and the ability to perform in situ remediation [3,4,5]. Plants and fungi are recognized as primary sources for both biofortification with Se as well as bioremediation [6,7,8].

Mushrooms are widely recognized for their diversity and nutritional value. Moreover, they possess the ability to uptake and convert inorganic Se into organic Se, with their capacity for Se accumulation varying depending on strain resources, culture conditions, source, and dosage of Se compounds. Given their bioaccumulative potential, mushrooms have been extensively studied and improved for both environmental and nutritional applications. In terms of environmental studies, mushrooms can serve as promising bioremediators for Se-induced pollution [9]. Hyperaccumulators possess the capability to accumulate elements at exceedingly high concentrations without causing toxicity [10], thereby exhibiting significant potential for food production, agriculture, and environmental applications. The acquisition of Se tolerance may be a prerequisite for hyperaccumulation [11]. The underlying mechanisms of Se tolerance and accumulation in typical plants have been the subject of several reports, highlighting key genes associated with transport, defense, and oxidative stress responses [12,13]. Metabolites such as flavonoids, phenylpropanoids, and selenolanthionine enhance plants’ tolerance towards Se [14,15,16]. Additionally, cell walls play a crucial role in the sequestration of selenate within vacuoles [15,17]. However, the mechanism underlying selenite tolerance and bioaccumulation in mushrooms remains unclear.

Several mushroom species, including *Flammulina velutipes* [18], *Hypsizygus marmoreus* [19], *Lentunula edodes* [20], *Pleurotus* [21], and *Ganoderma lucidum* [22], have been identified as effective candidates for Se biotransformation and bioremediation. However, the tolerance capacity of mushrooms towards Se varies with species. High levels of Se can pose a challenge for extremely polluted environments where mushrooms cannot grow normally. Although *G. lucidum* has a high accumulation ability for Se, it still exhibits toxic phenotypes at certain concentrations. In our previous studies, we found that *G. lucidum* CGMCC 5.26 was intolerant to 200 mg/kg selenite in liquid culture and exhibited poisoning phenotypes such as red coloration, pungent smell and decreased biomass [23].

To elucidate the mechanism behind selenite tolerance, *G. lucidum* JNUSE-200 with enhanced selenite tolerance was screened and obtained by laboratory adaptive evolution from *G. lucidum* CGMCC 5.26. Physiological and molecular responses to high selenite levels were compared in *G. lucidum* CGMCC 5.26 and *G. lucidum* JNUSE-200. Transcriptome-wide difference analysis revealed gene expression changes in both strains under control and selenite-cultured conditions while identifying key genes conferring selenite tolerance on a large scale, and it also elucidated the metabolic adaption of *G. lucidum* JNUSE-200 under selenite stress. This present study deepens the understanding of fungal tolerance towards selenite, thereby benefiting bioprocesses involving fungal selenite transformation and offering theoretical guidance for its application in bioremediation processes.

## 2. Materials and Methods

### 2.1. Strains and Culture Conditions

*G. lucidum* CGMCC 5.26 was purchased from the China General Microbiological Culture Collection Center (CGMCC) and preserved on potato dextrose agar slants at 4 °C. *G. lucidum* JNUSE-200 was obtained by laboratory adaptive evolution with selenite culture and preserved on potato dextrose agar (PDA) slants at 4 °C. 

The seed liquid culture medium was composed of glucose (20 g/L), yeast nitrogen base without amino acids (5 g/L), tryptone (5 g/L), KH_2_PO_4_ (4.5 g/L), and MgSO_4_·7H_2_O (2 g/L) at the initial pH. The fermentation liquid culture medium was composed of glucose (20 g/L), yeast nitrogen base without amino acids (5 g/L), tryptone (5 g/L), KH_2_PO_4_ (4.5 g/L), MgSO_4_·7H_2_O (2 g/L), and 200 mg/kg Na_2_SeO_3_ at the initial pH. 

### 2.2. Laboratory Adaptive Evolution

Enhanced selenite-tolerant strains of *G. lucidum* were continuously screened through adaptive evolution using a gradient concentration of Na_2_SeO_3_ in solid PDA plates and liquid culture derived from the original *G. lucidum* CGMCC 5.26 strain. The initial Na_2_SeO_3_ concentration (200 mg/kg) was chosen based on the strain’s maximum tolerance to these conditions. The concentration of Na_2_SeO_3_ was increased twofold after 10 generations of culture. For nearly two years, a *G. lucidum* strain capable of tolerating 200 mg/kg Na_2_SeO_3_ in liquid culture was selected and designated as *G. lucidum* JNUSE-200, which has been preserved at the China Center for Type Culture Collection (CCTCC).

### 2.3. Biomass, Extracellular Polysaccharides (EPS) and Intracellular Polysaccharides (IPS) 

The mycelia were obtained by centrifugation at 10,000 rpm for 10 min, followed by three washes with distilled water and subsequent lyophilization. The biomass was determined at room temperature using a gravimetric method.

Extracellular polymeric substances (EPS) and internal polymeric substances (IPS) were quantified using the phenol-sulfuric acid method [22]. EPS was precipitated from the fermentation supernatant with 95% (*v*/*v*) ethanol (4 times), then stored at a temperature of 4 °C for 8 h. Crudes were obtained through centrifugation, washed three times with 75% (*v*/*v*) ethanol, and further dried at room temperature to eliminate residual ethanol. They were subsequently dissolved in water, and their concentrations were determined. Mycelia powder weighing 20.0 mg was suspended in 10 mL H_2_O at a temperature of 100 °C for a duration of 3 h. The resulting supernatant was collected via centrifugation and its volume was recorded. The same method used for extracting EPS from the supernatant was employed for obtaining IPS.

### 2.4. Se Accumulation Capacity Determination

The total Se accumulated in the mycelia was measured using an inductively coupled plasma mass spectrometer (ICP-MS; Agilent 7700, Agilent Technologies, Waldbronn, Germany) [23]. The bioconcentration factor (BCF) was used to compare the Se accumulation efficiency. The BCF was defined as the ratio of the concentration of Se accumulated in the mycelia to that in the corresponding media.

### 2.5. Macroscopic and Microscopic Morphology 

Macroscopic mycelial morphology was examined and imaged using a Nikon SMZ25 microscope (Tokyo, Japan). Microscopic mycelial cell ultrastructures were observed using a HITACHI H-7650 microscope (HITACHI, Tokyo, Japan) [22].

### 2.6. Selenite Uptake Kinetics

*G. lucidum* CGMCC 5.26 and *G. lucidum* JNUSE-200 were both cultured with selenite concentrations ranging from 25 to 200 mg/kg in liquid media on the 4th day. The samples were obtained by centrifugation, cleaned three washes with deionized water, and frozen. The total Se content was determined by ICP-MS.

### 2.7. X-ray Photoelectron Spectroscopy (XPS) 

XPS detection was conducted using an AXIS Supra spectrometer by Kratos Analytical Inc. (Manchester, UK), equipped with monochromatized Al Kα radiation (hv = 1486.6 eV, 150 W) as the X-ray source, and operated under a base pressure of 10-9 Torr. Survey scan spectra were acquired with a pass energy of 160 eV and a step size of 1 eV. Narrow-region scans were obtained with a pass energy of 40 eV and a step size of 0.1 eV. The hybrid lens mode was employed in both cases. The examined area for XPS spectra was set at 300 × 700 µm^2^. A charge neutralizer was consistently used throughout the detection process to ensure electrical isolation from the sample bar. All spectra were calibrated using C 1 s (284.8 eV).

### 2.8. Transcriptome Profiling Analysis

Mycelia cultured with and without 200 mg/kg selenite were collected on the 4th, 5th, 6th and 8th day for transcriptome sequencing. Three biological replicates were prepared for each experiment. RNA extraction, library construction, sequencing, and data processing were conducted with the help of the Applied Protein Technology Company (Shanghai, China). Differentially expressed genes (DEGs) between the two comparative samples were identified using the DEseq2 package (1.10.1), with a *p* value (FDR) < 0.05, and |log2 (fold change)| > 1 was set as the cut-off for identifying DEGs. Enrichment analysis of the DEGs was performed using GO and KEGG databases to obtain a detailed description of the DEGs. Principal component analysis (PCA) was used to assess differences between groups.

The samples used in this study were *G. lucidum* CGMCC 5.26 cultured without selenite (C-0), *G. lucidum* CGMCC 5.26 cultured with 200 mg/kg selenite (C-200), *G. lucidum* JNUSE-200 cultured without selenite (H-0), and *G. lucidum* JNUSE-200 cultured with 200 mg/kg selenite (H-200).

### 2.9. Gene Expression Analysis by RT-qPCR

The Plant/Fungi Total RNA Purification Kit (Norgen Biotek Corp., Thorold, ON, Canada) was used for RNA extraction. RNA quality and quantity were measured with a Nanodrop ND-1000 spectrophotometer (Nanodrop Technologies, Wilmington, DE, USA). RNA samples were stored at −80 °C.

RT-qPCR was performed to confirm the results of the RNA-Seq analysis. cDNA was synthesized from extracted total RNA using a GoTaq^®^ 2-Step RT-qPCR kit (PROMEGA, Madison, WI, USA). PCR reactions were carried out in the LightCycler^®^480 Instrument II (Roche, Basel, Switzerland). Four transcripts were selected for validation. Transcript-specific primers were designed using BlastPrimer +2.15.0 (Supplementary Table S1). Three biological and technical replicates were performed for both the control and stress samples. RNS was used as the internal reference gene for normalization. Relative changes in expression levels were calculated using the 2^−∆∆CT^ method [24]. 

### 2.10. Statistical Analysis

Data were presented as the mean ± standard deviation (SD) from three replicates for each assay. Statistical significance was analyzed by one-way analysis of variance (ANOVA) using SPSS 20.0 software. Comparison with *p* < 0.05 was statistically significant.

## 3. Results and Discussion

### 3.1. G. lucidum JNUSE-200 Exhibited Enhanced Selenite Tolerance

**Figure 1:**
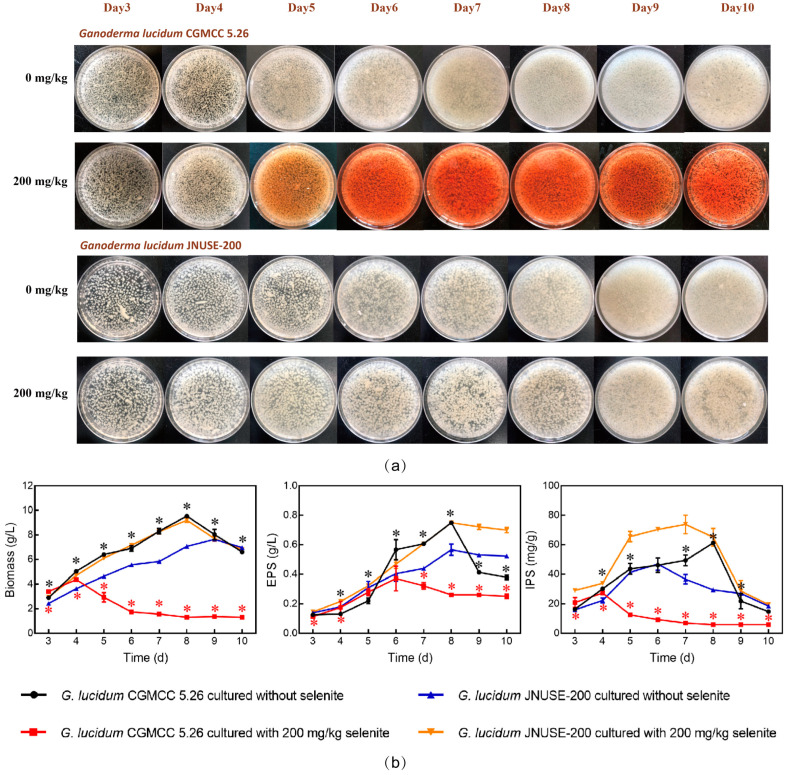
(**a**) The growth phenotype of *G. lucidum* CGMCC 5.26 and *G. lucidum* JNUSE-200 in liquid culture; (**b**) biomass, EPS and IPS of *G. lucidum* CGMCC 5.26 and *G. lucidum* JNUSE-200. * indicates a significant difference at *p* < 0.05 between *G. lucidum* CGMCC 5.26 and *G. lucidum* JNUSE-200 cultured without selenite; * indicates a significant difference at *p* < 0.05 between *G. lucidum* CGMCC 5.26 and *G. lucidum* JNUSE-200 cultured with 200 mg/kg selenite.

Selenite tolerance is defined as the ability to survive and grow normally at high levels of Se. The growth status of the two *Ganoderma* spp. was investigated during liquid culture. During the 10-day culture of *G. lucidum* CGMCC 5.26 grown in 200 mg/kg selenite, symptoms became apparent from the fifth day, as indicated by red mycelia, a pungent odor, and declining biomass. However, *G. lucidum* JNUSE-200 growth was stimulated with no signs of toxicity and displayed the same phenotype as *G. lucidum* JNUSE-200 grown without selenite (Figure 1a). Therefore, *G. lucidum* JNUSE-200 exhibited enhanced selenite tolerance compared with *G. lucidum* CGMCC 5.26.

Selenite stress adversely affected *G. lucidum* CGMCC 5.26 growth but positively influenced *G. lucidum* JNUSE-200 growth. The addition of selenite resulted in an increasing trend in biomass for *G. lucidum* JNUSE-200 while causing a decreasing trend in biomass for *G. lucidum* CGMCC 5.26. Specifically, *G. lucidum* JNUSE-200 cultured with 200 mg/kg selenite accumulated a biomass similar to that of *G. lucidum* CGMCC 5.26 cultured without selenite (Figure 1b). The maximal specific growth rate of G. lucidum JNUSE-200 mycelia was 0.467 d^−1^, while the growth rate of *G. lucidum* CGMCC 5.26 exhibited negative values as a result of the detrimental impact caused by high concentrations of sodium selenite (Supplementary Figure S1). Thus, it could be concluded that *G. lucidum* JNUSE-200 was completely tolerant to, and even benefited from, 200 mg/kg selenite, while *G. lucidum* CGMCC 5.26 was sensitive to and negatively affected by the same concentration of selenite.

As an important bioactive metabolite of *G. lucidum*, polysaccharides were used to explore the effects of selenite on secondary metabolism. Selenite application affected the polysaccharides in both *G. lucidum* CGMCC 5.26 and *G. lucidum* JNUSE-200. In particular, 200 mg/kg selenite increased the *G. lucidum* JNUSE-200 extracellular polysaccharide (EPS) and intracellular polysaccharide (IPS) yields and decreased the IPS and EPS yields in *G. lucidum* CGMCC 5.26 (Figure 1b). Selenite stress adversely affects the growth and productivity of plants and microorganisms. It is generally believed that at the same concentration, Se can promote the growth of Se-accumulating plants but has toxic effects on non-Se-accumulating plants. These findings indicated that the highly selenite-tolerant *G. lucidum* JNUSE-200 relied on selenite to promote the growth and production of major bioactive metabolites.

### 3.2. G. lucidum JNUSE-200 Exhibited Macroscopic and Microscopic Morphological Differences

**Figure 2:**
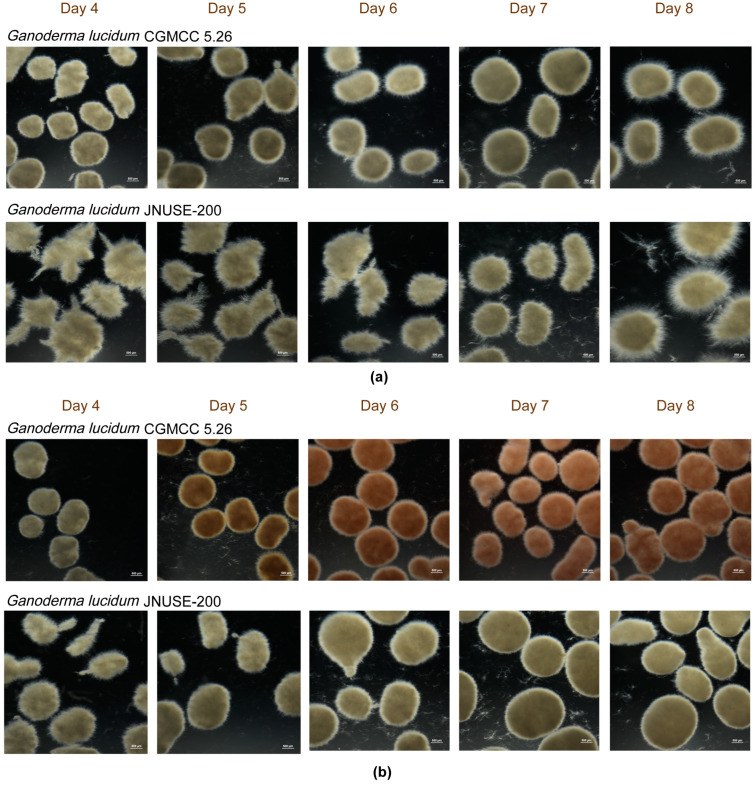
The apparent forms of *G. lucidum* CGMCC 5.26 and *G. lucidum* JNUSE-200 mycelium pellets (**a**) cultured without selenite and (**b**) cultured with 200 mg/kg selenite.

There were also significant macro-morphological differences observed between the two strains. In the absence of selenite, *G. lucidum* CGMCC 5.26 formed compact mycelia pellets, while *G. lucidum* JNUSE-200 exhibited feathery, flake-like and irregular growth patterns before forming rough-edged pellets that lacked internal density or compactness (Figure 2a). In the presence of selenite, *G. lucidum* CGMCC 5.26 displayed a color change from white to orange and red, with loose and easily breakable mycelia formations. On the other hand, *G. lucidum* JNUSE-200 gradually transformed from irregular sheets into tightly packed pellets with smooth edges and dense interiors (Figure 2b). The clustering of *G. lucidum* JNUSE-200 mycelia into pellets was dependent on selenite stimulation for growth as it required selenite for this process to occur effectively at high concentrations of selenite levels.

**Figure 3:**
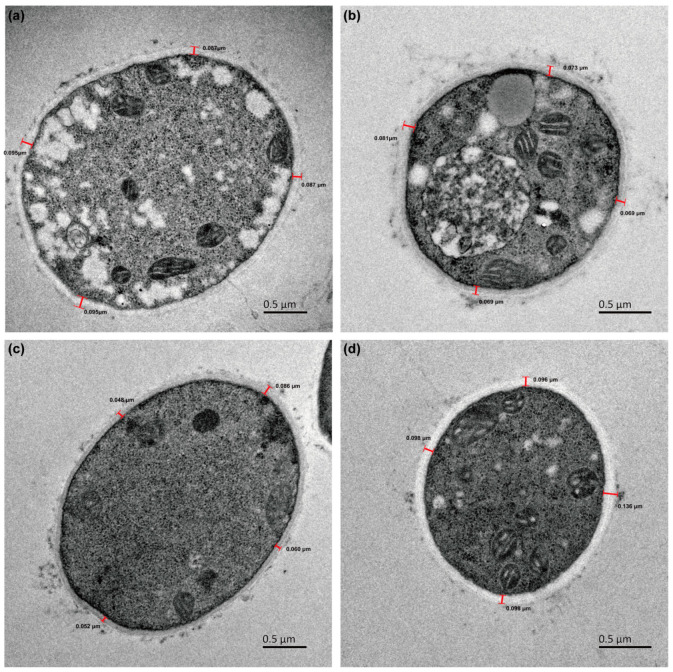
The *G. lucidum* CGMCC 5.26 and *G. lucidum* JNUSE-200 cell wall thicknesses cultured without selenite on the 4th day (**a**,**c**) and 8th day (**b**,**d**).

Moreover, there were variations in micro-morphology particularly in terms of cell walls among the mycelial cells, which play a crucial role in pellet morphology and changes related to assimilation of selenite by fungi cells. Transmission electron microscopy (TEM) analysis revealed that without exposure to selenite, the cell wall thicknesses decreased over time for *G. lucidum* CGMCC 5.26 from 0.089 ± 0.006 μm on the fourth day to 0.073 ± 0.006 μm on the eighth day; for *G. lucidum* JNUSE-200, they increased from 0.062 ± 0.017 μm on the fourth day to 0.107 ± 0.019 μm on the eighth day, respectively (Figure 3).

Morphological engineering has recently been proposed as a novel strategy for constructing efficient microbial cell factories to control cell shape and division patterns [25]. There were significant differences in the macroscopic mycelial morphology and microscopic cell structures between *G. lucidum* JNUSE-200 and *G. lucidum* CGMCC 5.26. This indicates that during the adaptive evolution process, *G. lucidum* JNUSE-200 could effectively counteract negative effects associated with elevated levels of selenite by enhancing its cell wall thickness.

### 3.3. Total Se Accumulated in G. lucidum CGMCC 5.26 and G. lucidum JNUSE-200

**Figure 4:**
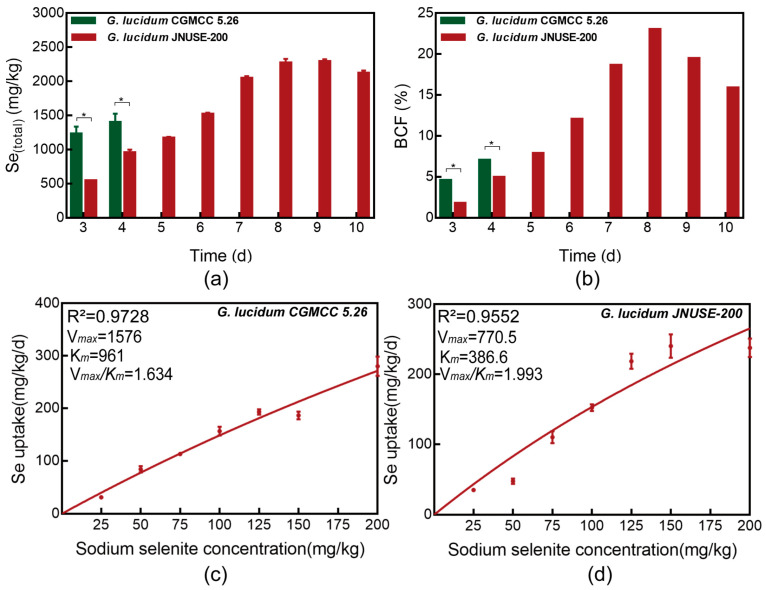
*G. lucidum* CGMCC 5.26 and *G. lucidum* JNUSE-200 (**a**) Se accumulation capacity and (**b**) BCF value, and the kinetics of selenite influx to the mycelia of (**c**) *G. lucidum* CGMCC 5.26 and (**d**) *G. lucidum* JNUSE-200 within 4 days of exposure to different selenite concentrations. * indicates a significant difference at *p* < 0.05.

Accumulators were classified based on the amounts of elements they accumulated or their capacity to accumulate elements under a normal growth status [13]. These Se levels in *G. lucidum* confirmed that this specie was a Se hyper-accumulator, with the maximum Se accumulated in *G. lucidum* CGMCC 5.26 and *G. lucidum* JNUSE-200 cultured with 200 mg/kg selenite being 1398.41 mg/kg and 2290.06 mg/kg in liquid culture (Figure 4a). Moreover, compared to *G. lucidum* CGMCC 5.26, *G. lucidum* JNUSE-200 showed a decreased selenite uptake on the 4th–5th day.

The Se BCF values in *G. lucidum* JNUSE-200 mycelia increased during the liquid culture process, reaching a maximum value of 23.01% on the eighth day; for *G. lucidum* CGMCC 5.26, the BCF values were under 10% (Figure 4b).

### 3.4. Uptake Dynamics of Selenite in G. lucidum CGMCC 5.26 and G. lucidum JNUSE-200

The selenite uptake rate varied between *G. lucidum* CGMCC 5.26 and *G. lucidum* JNUSE-200. The influx of selenite into the mycelia could be accurately described by the Michaelis–Menten equation, and linear equations were fitted to the data with regression coefficients of 0.9728 and 0.9552, respectively (Figure 4c,d). *G. lucidum* CGMCC 5.26 exhibited higher Vmax and Km values compared to *G. lucidum* JNUSE-200, indicating a greater capacity for selenite uptake in *G. lucidum* CGMCC 5.26 than in *G. lucidum* JNUSE-200. The Vmax of selenite uptake decreased by 51% in *G. lucidum* JNUSE-200, suggesting that it has a lower efficiency in absorbing selenite compared to *G. lucidum* CGMCC 5.26. *G. lucidum* JNUSE-200 also demonstrated a higher affinity for selenite than *G. lucidum* CGMCC 5.26. Furthermore, the substrate specificity for selenite was calculated from Vmax/Km, and *G. lucidum* JNUSE-200 displayed a higher specificity for selenite than *G. lucidum* CGMCC 5.26. This observation is consistent with Se-hyperaccumulating plants, which preferentially take up Se [26].

### 3.5. XPS Analysis of Se in G. lucidum JNUSE-200 Mycelia

**Figure 5:**
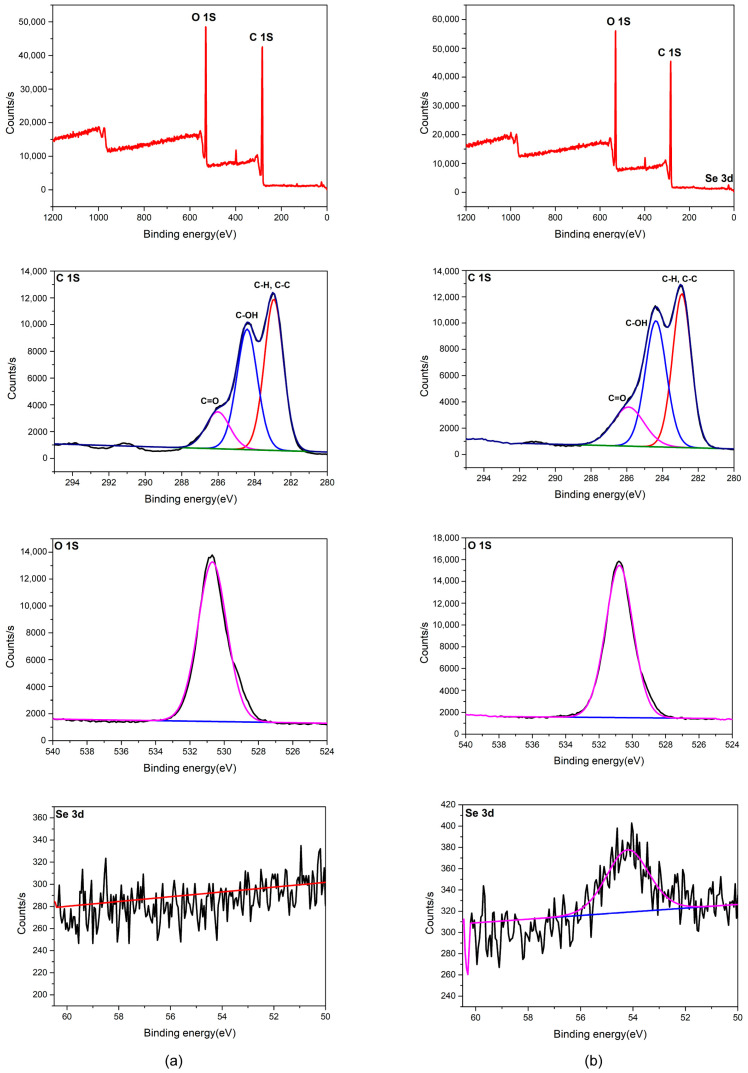
XPS spectra of the *G. lucidum* JNUSE-200 mycelia (**a**) cultured without selenite and (**b**) cultured with 200 mg/kg selenite. The red curve represents a high-resolution XPS full spectrum; The black line represents the original XPS spectra of element C, O and Se; and the color line represents a finely-tuned fitting curve of the C, O and Se.

The X-ray photoelectron spectroscopy (XPS) analysis of *G. lucidum* JNUSE-200 mycelia cultured without and with 200 mg/kg selenite on the eighth day is presented in Figure 5. The control mycelia exhibited two prominent elemental peaks, namely O at 531.12 eV and C at 283.38 eV. The C1S spectrum was fitted into three components at 286.39 (14.18%), 284.78 (39.94%) and 283.31 (45.88%) eV, corresponding to C=O, C-OH, C-H and C-C bonds respectively. The O-1s spectrum appeared at a binding energy of 531.12 eV (100%). The XPS spectra did not include any entries for selenium.

Similarly, the XPS spectra of the cultured mycelia also exhibited two prominent elemental peaks: O at 531.15 eV and C at 283.37 eV. The fitting analysis of the C1S spectrum revealed three distinct components located at binding energies of 286.37 (14.70%), 284.74 (43.56%), and 283.28 (41.73%) eV, respectively. A weaker signal at 283.28 eV was attributed to a reduction in C-H and C-C bonds, while a stronger signal at 284.74 eV indicated an increase in C-OH bonds, suggesting that substitution by hydroxyl groups occurred. The O-1s spectrum appeared at a binding energy of 531.15 eV (100%). In particular, a newly formed Se3d peak centered at 54.58 eV was observed in the survey spectra corresponding to selenide species, demonstrating that *G. lucidum* JNUSE-200 actively converted selenite (Ⅳ) into bioavailable selenides (-Ⅱ) [27].

### 3.6. Transcriptome Analysis of G. lucidum CGMCC 5.26 and G. lucidum JNUSE-200 in Response to Selenite

**Figure 6:**
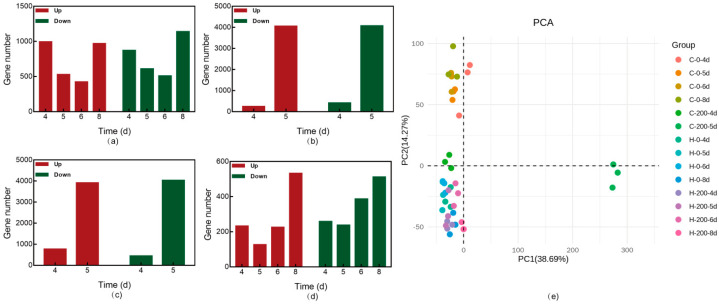
DEGs in *G. lucidum* CGMCC 5.26 and *G. lucidum* JNUSE-200: (**a**) C-0 vs. H-0, (**b**) C-200 vs. H-200, (**c**) C-0 vs. C-200 and (**d**) H-0 vs. H-200; (**e**) two−dimensional spatial distribution of PCA principal components. Note: C-0: *G. lucidum* CGMCC 5.26 cultured without selenite; C-200: *G. lucidum* CGMCC 5.26 cultured without selenite; H-0: *G. lucidum* JNUSE-200 cultured without selenite; H-200: *G. lucidum* JNUSE-200 cultured without selenite.

A comprehensive understanding of the molecular mechanisms underlying selenite tolerance and accumulation is essential for fully harnessing the potential of fungi as bioremediation sources. Therefore, *G. lucidum* CGMCC 5.26 and *G. lucidum* JNUSE-200 were cultured in liquid medium with 200 mg/kg selenite, while a control group was cultured without selenite. Forty-two cDNA libraries were prepared from three replicates per selenite culture condition and sequenced to gain insights into the transcriptome of *G. lucidum* and identify key genes responsive to selenite (Supplementary Table S2). Figure 6a–d provides an overview of gene expression changes in response to selenite exposure.

Pairwise comparison analysis was conducted between *G. lucidum* CGMCC 5.26 and *G. lucidum* JNUSE-200 (C-0 vs. H-0 and C-200 vs. H-200), as well as between control samples and those cultured with selenite within each strain (C-0 vs. C-200 and H-0 vs. H-200). Under the conditions of culturing with 200 mg/kg selenite, there was a significantly lower number of differentially expressed genes (DEGs) in *G. lucidum* JNUSE-200 compared to *G. lucidum* CGMCC 5.26, indicating that *G. lucidum* CGMCC 5.26 exhibited higher sensitivity towards elevated levels of selenite, requiring more genetic responses to mitigate its negative impact on mycelial growth.

Principal component analysis (PCA) based on DEGs revealed distinct clustering patterns for *G. lucidum* CGMCC 5.26 and *G. lucidum* JNUSE-200 in two-dimensional spatial areas (Figure 6e), suggesting that these two strains displayed differences at the genetic level during their adaptive evolution process, resulting in inconsistent growth phenotypes for *G. lucidum* JNUSE-200 and enhanced tolerance towards selenite.

#### 3.6.1. Modulation Selenite Tolerance via the Cell Wall 

The cell wall is a vital extracellular structure in fungi that provides protection against osmotic pressure and mechanical forces. As an important core component of the cell wall, chitin content varies greatly among different species, and the chitin content of the same species also changes with the physiological state. Chitin synthase is a key enzyme involved in chitin synthesis [28]. Compared to *G. lucidum* CGMCC 5.26, *G. lucidum* JNUSE-200 upregulated the expression of putative chitin synthase 7, 5, 2, and 3 genes, particularly putative chitin synthase 5 and chitin synthase 7 genes, which may regulate selenite absorption and enhance selenite tolerance by increasing cell wall thickness. Furthermore, there were distinct expression patterns observed for five fungal hydrophobin genes between *G. lucidum* JNUSE-200 and *G. lucidum* CGMCC 5.26 (Supplementary Table S3). Notably, the expression levels of two fungal hydrophobin genes, GL30893-G and GL24177-G, were higher in *G. lucidum* JNUSE-200 compared to those in *G. lucidum* CGMCC 5.26. The expression levels of hydrophobins GL16371-G and GL15943-G were increased by selenite treatment in *G. lucidum* JNUSE-200. Fungal hydrophobins coat the fungal surface and interact with the chitinous cell wall closely related to hydrophobicity while influencing the carbohydrate type distribution, thus protecting cell wall integrity. Fungal hydrophobins have been reported to play a role in heat stress resistance, protecting fungi from harsh environments [29].

Currently, no reports have been published on the correlation between morphology and selenite tolerance. However, a few studies have reported a correlation between plant morphology and tolerance towards environmental factors such as cold [30] and salt [31]. Therefore, it is of high practical significance to investigate novel or expanded parameters to depict micro-morphological and physiological characteristics associated with selenite tolerance in plants and microorganisms. This may pave new avenues for elucidating critical mechanisms.

#### 3.6.2. Modulation Selenite Tolerance via Membrane Channels

The regulation of transporters involved in selenite transportation is presumably responsible for Se accumulation in *G. lucidum*. Based on studies on Se metabolism in plants, inorganic Se is transported to cells by sulfate and phosphate transporters [26]. Therefore, the transcript levels of putative sulfur (S) and phosphate (P) transporter genes were analyzed.

In the absence of selenite, the expression levels of sulfite efflux pump genes (GL25356-G/GL19873-G) and inorganic P transporter genes (GL23411-G) in *G. lucidum* JNUSE-200 were significantly higher than in *G. lucidum* CGMCC 5.26. In the presence of selenite, the sulfite efflux pump gene (GL19873-G), two sulfate transporter genes, and five inorganic P transporter genes were differentially expressed in *G. lucidum* CGMCC 5.26 and *G. lucidum* JNUSE-200; *G. lucidum* JNUSE-200 upregulated six transporter genes and downregulated two transporter genes (Supplementary Table S4). Moreover, the expression of permease genes in *G. lucidum* CGMCC 5.26 and *G. lucidum* JNUSE-200 was largely discrepant with and without selenite. In particular, the expression of the high-affinity methionine permease gene (GL20912-G) in *G. lucidum* JNUSE-200 was higher than that in *G. lucidum* CGMCC 5.26, regardless of the addition of selenite. 

The sulfite efflux pump, which belongs to the Tellurite-resistance/Dicarboxylate Transporter (TDT) family, binds to the cell membrane and regulates sulfite excretion [32], and its expression is associated with selenite tolerance in Saccharomyces cerevisiae [33,34]. Met is an essential amino acid required for a variety of processes in organisms; however, excess Met drives proteotoxicity [35]. The high-affinity methionine permease gene is a key gene for growth and amino acid transport, particularly that of Met. The raised expression could expel excess Met from the cell and prevent its non-specific metabolism to SeMet, so the high-affinity methionine permease gene is important for its potential to minimize or eliminate Met and SeMet toxicity in *G. lucidum* JNUSE-200. Plants take up selenite via phosphate transporters [36], and membrane phosphate transporters are vital for enhancing selenite tolerance in well-studied plants. Increased expression of the phosphate transporter gene OsPT8 improves Se content in *Nicotiana tabacum* [37]. In *Arabidopsis thaliana*, terpenoid synthase (TPS22) mediates Se tolerance through the reduction in Se absorption and activation of metabolic detoxification, which decreases the expression of high-affinity transporters PHT1;1, PHT1;8, and PHT1;9 [38]. Therefore, we considered that the high expression of the sulfite efflux pump, inorganic P transporter, and high-affinity methionine permease genes might exert a direct influence on mediating *G. lucidum* JNUSE-200 selenite tolerance under high selenite conditions. 

#### 3.6.3. Modulation Selenite Tolerance via Internal Metabolic Occurrence

Identifying the metabolic pathways directly influenced by selenite remains a major challenge in exploring selenite tolerance and accumulation. KEGG pathway analyses revealed that genes involved in Se compound metabolism (Supplementary Table S5), sulfur metabolism (Supplementary Table S6), metabolism of the xenobiotics pathway (Supplementary Tables S7–S9), and antioxidant system (Supplementary Table S10) exhibited distinct expression patterns between *G. lucidum* CGMCC 5.26 and *G. lucidum* JNUSE-200.

(1) Se compound metabolism

**Figure 7:**
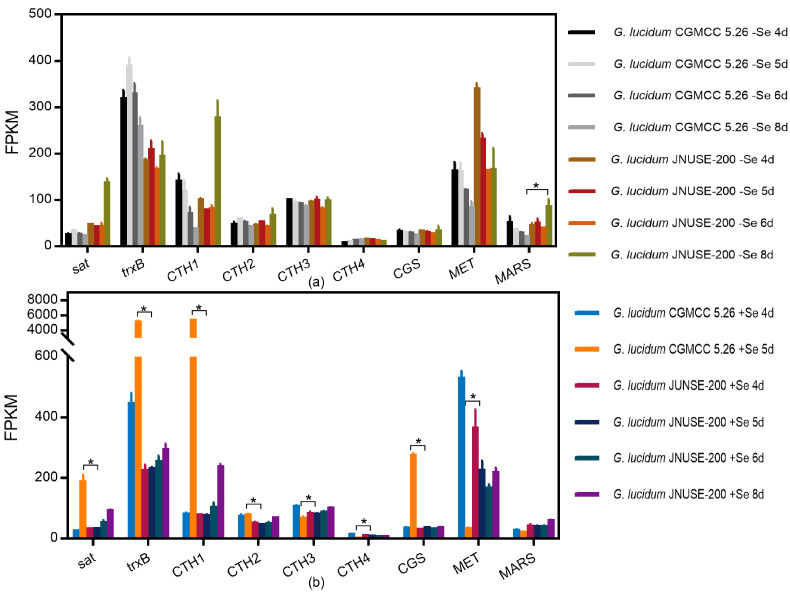
FPKM values of Se metabolism pathway genes. * indicates a significant difference at *p* < 0.05. (**a**) C-0 vs. H-0, (**b**) C-200 vs. H-200. Note: C-0: *G. lucidum* CGMCC 5.26 cultured without selenite; C-200: *G. lucidum* CGMCC 5.26 cultured without selenite; H-0: *G. lucidum* JNUSE-200 cultured without selenite; H-200: *G. lucidum* JNUSE-200 cultured without selenite.

In *G. lucidum* CGMCC 5.26 and *G. lucidum* JNUSE-200 cultured without selenite, only *G. lucidum* JNUSE-200 increased the attachment of selenomethionine to tRNA (Met) on the eighth day by increasing the methionyl-tRNA synthetase (MARS) gene expression by 1.753-fold (Figure 7a).

In *G. lucidum* CGMCC 5.26 and *G. lucidum* JNUSE-200 cultured with 200 mg/kg selenite on the fifth day, *G. lucidum* JNUSE-200 prominently down-regulated the gene expression levels of cystathionine gamma-lyase 1,2 and 3 (CTH 1,2,3), thioredoxin reductase (NADPH) (trxB), and 3′-phosphoadenosine 5′-phosphosulfate synthase (sat) by 7.614-, 2.239-, 1.215-, 5.948-, and 3.991-fold, respectively, and upregulated homocysteine methyltransferase (MET) and cystathionine gamma-synthase (CGS) by 1.308- and 1.152-fold, respectively (Figure 7b). 

In comparison with *G. lucidum* CGMCC 5.26 cultured without selenite, 200 mg/kg of selenite-cultured *G. lucidum* CGMCC 5.26 showed normal growth on the fourth day, and only MARS gene expression was upregulated; on the fifth day (abnormal growth), five upregulated genes and one downregulated gene in the Se compound metabolism pathway were identified. For *G. lucidum* JNUSE-200, mycelia grew normally under the two culture conditions throughout the process, and no DEGs in the Se compound metabolism pathway were identified for *G. lucidum* JNUSE-200.

Selenocysteine (SeCys) and selenomethionine (SeMet) can nonspecifically replace cysteine (Cys) and methionine (Met) in proteins, leading to protein dysfunction and toxicity [39]. Non-specific accumulation of SeMet is found to be less adverse than that of SeCys [40]. Puccinellia distans increases Se by decreasing the expression of methionyl-tRNA synthetase and cystathionine gamma-lyase to inhibit the nonspecific incorporation of Se into proteins [17]. The formation of organic MeSeCys and volatile Se species contributes to Se tolerance and hyperaccumulation in Cardamine enshiensis [41], and our results confirmed that selenation is an important mechanism for Se detoxification [42]. In the condition of selenite culture, compared to *G. lucidum* CGMCC 5.26, *G. lucidum* JNUSE-200 increased CGS and MET gene expression, enhancing the transformation of SeCys to SeMet, and *G. lucidum* JNUSE-200 decreased the gene expression of CTH 1,2,3 and trxB, maintaining methyl-selenocysteine (MeSeCys) and methyl-selenomethionine (MeSeMet) in the form of a methyl compound, consequently decreasing the amount of available SeCys and SeMet for incorporation into proteins [43]. These gene expression changes indicated that 200 mg/kg selenite had no negative effects on *G. lucidum* JNUSE-200, and it was not necessary to activate the expression of these genes to overcome adversity. *G. lucidum* CGMCC 5.26 needed to activate the high expression of these genes to transform selenite to a lower valence state of Se, reducing its toxic effect (Supplementary Figure S2) [44].

(2) Sulfur metabolism

**Figure 8:**
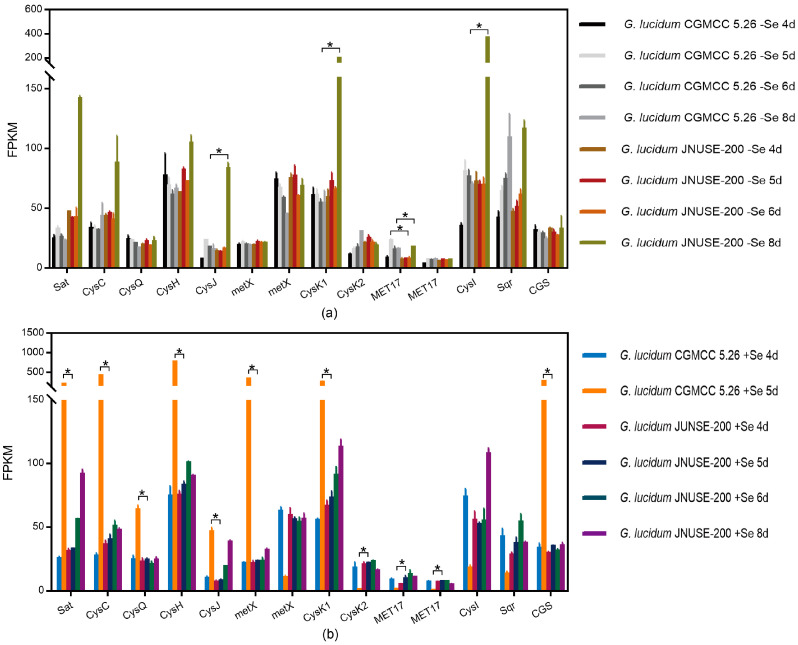
FPKM values of sulfur metabolism pathway genes. * indicates a significant difference at *p* < 0.05. (**a**) C-0 vs. H-0, (**b**) C-200 vs. H-200. Note: C-0: *G. lucidum* CGMCC 5.26 cultured without selenite; C-200: *G. lucidum* CGMCC 5.26 cultured without selenite; H-0: *G. lucidum* JNUSE-200 cultured without selenite; H-200: *G. lucidum* JNUSE-200 cultured without selenite.

As shown in Figure 8, the transcript levels of S metabolism pathway genes differed greatly between the two *G. lucidum* species. Notably, the application of selenite affected S metabolism in *G. lucidum* CGMCC 5.26, whereas for *G. lucidum* JNUSE-200, only the transcript levels of CysI (sulfite reductase (NADPH) hemoprotein subunit) and CysJ (sulfite reductase flavoprotein subunit) genes were downregulated by selenite.

Compared to *G. lucidum* CGMCC 5.26 cultured without selenite, *G. lucidum* JNUSE-200 downregulated MET17 (*O*-acetylhomoserine/*O*-acetylserine sulfhydrylase) expression at the 5th–6th day by 1.639- and 1.016-fold, respectively, while CysI, CysJ, and CysK (cysteine synthase 1) genes were downregulated much more significantly, with Log2 (fold change) values of 1.639, 1.744 and 1.258 on the eighth day. 

In comparison with *G. lucidum* CGMCC 5.26 cultured with 200 mg/kg selenite, *G. lucidum* JNUSE-200 downregulated the expression of sat, CysC (adenylyl-sulfate kinase), CysQ (3′(2′), 5′-bisphosphate nucleotidase), CysH (phosphoadenosine phosphosulfate reductase), metB (cystathionine gamma-synthase), CysJ, CysK1 and metX (homoserine *O*-succinyltransferase/*O*-acetyltransferase) genes, and upregulated MET17 and CysK2 expression (Supplementary Figure S3).

One reason for Se toxicity in plants is its interference with sulfur (S) metabolism, leading to the formation of Se analogs in S-containing organic compounds. The incorporation of Se into proteins results in altered protein structures and ultimately leads to metabolic abnormalities. Se hyperaccumulators can tolerate high levels of Se by either preventing the incorporation of Se into proteins or enhancing the repair capabilities of incorporated proteins [12,45,46]. Addressing the interactions between Se and S is crucial for understanding Se toxicity [47]. In the hyperaccumulator *Stanleya pinnata*, selenate uptake was less affected by high-S pretreatment compared to non-hyperaccumulators *Stanleya elata* and *Brassica juncea* [48]. Active *O*-acetylserine-(thiol) lyases A and B degrade l-Cys and l-SeCys while conferring improved resistance against Se in *Arabidopsis* [49]. CpNifS catalyzes the conversion of both Cys into alanine and elemental S, as well as converting SeCys into alanine and elemental Se; increased expression of AtCpNifS enhances tolerance towards and accumulation of Se in *Arabidopsis* [50]. These results demonstrated that the S metabolism pathway was activated in large quantities only when selenite caused growth inhibition and toxicity to mycelia. Sulfite reductases CysJ and CysI catalyze the reduction of sulfite/selenite to sulfide/selenide, whereas CysK catalyzes the reduction from sulfide/selenide to Cys/SeCys. Finally, toxic selenite is reduced to selenide by sulfite reductase. However, regardless of the addition of selenite, differences were observed in gene expression patterns for CysJ and CysI: *G. lucidum* JNUSE-200 showed decreased expression with selenite culture while *G. lucidum* CGMCC 5.26 exhibited increased expression with selenite culture. These findings indicated that enhanced resistance against selenite mainly depend on different gene expression patterns for CysJ and CysI, which regulate synthesis pathways for both Cys and SeCys, thereby regulating their non-specific incorporation into proteins.

(3) Metabolism of xenobiotics

To cope with environmental stress and regulate high selenite levels, various gene families are involved in sensing and responding to such stress, including cytochrome P450 (CYP450), glutathione S transferase (GSTs), and ATP-binding cassette (ABC) transporter gene families. These gene families play a role in modifying, binding, and secreting exogenous substances [51,52,53]. The expression of CYP450, GSTs, and ABC transporter superfamily genes was specifically induced by selenite, indicating their involvement in abiotic stress resistance in *G. lucidum* (Supplementary Tables S7–S9).

In the comparison of gene expression between *G. lucidum* CGMCC 5.26 and *G. lucidum* JNUSE-200, 162 CYP450 genes, 28 GSTs genes, and 32 ABC transporter genes were identified differently. A total of 56 CYP450 genes, 19 GSTs, and 8 ABC transporter genes were not induced differently by selenite in *G. lucidum* CGMCC 5.26 and *G. lucidum* JNUSE-200; 131 CYP450 genes, 22 GSTs genes, and 23 ABC transporter genes were induced differently by selenite in *G. lucidum* CGMCC 5.26 and *G. lucidum* JNUSE-200.

For *G. lucidum* CGMCC 5.26, the expression of 121 CYP450 genes, 23 GSTs genes, and 25 ABC transporter genes was found to differ with selenite addition, whereas for *G. lucidum* JNUSE-200 alone, only 41 CYP450 genes, 6 GSTs genes, and 9 ABC transporter genes were found to be altered with selenite addition. The metabolism of xenobiotic-related genes activated by *G. lucidum* JNUSE-200 was significantly lower than that of the original *G. lucidum* CGMCC 5.26, indicating that 200 mg/kg of selenite had a lower effect on *G. lucidum* JNUSE-200 than on *G. lucidum* CGMCC 5.26.

(4) Antioxidant defense system

The transcript of genes encoding antioxidant enzymes exhibited differential expression in *G. lucidum* CGMCC 5.26 (Supplementary Table S10). Elevated concentrations of selenite exerted a toxic effect on *G. lucidum* CGMCC 5.26, leading to the significant upregulation of antioxidant system genes, including manganese superoxide dismutase/superoxide dismutase/glutathione peroxidase (GPX)/ascorbate peroxidase (APX)/peroxin 7/peroxin 10, except for manganese peroxidase, which was downregulated by selenite exposure. Conversely, *G. lucidum* JNUSE-200 did not experience any negative impact or oxidative stress under the same concentration of selenite treatment, with no discernible alteration in the expression patterns of these enzyme-related genes observed, thus indicating that *G. lucidum* JNUSE-200 could fully tolerate this high selenite concentration without compromising its physiological state. Consequently, APX gene expression was found to be higher in *G. lucidum* JNUSE-200 compared to *G. lucidum* CGMCC 5.26 cultured without selenite and remained consistently elevated during the growth process, suggesting that adaptive evolution has endowed *G. lucidum* JNUSE-200 with enhanced antioxidant capacity. It is crucial for microorganisms to maintain a delicate balance between their antioxidant defense system and environmental stress. Defense-related genes were constitutively upregulated in Se hyperaccumulator plants; however, their direct mechanism of hyperaccumulation needs in-depth study [17].

#### 3.6.4. Transcription Factors

Transcription factors (TFs) play a crucial role in regulating the expression of multiple genes involved in the stress response mechanism, enabling plants and microorganisms to tolerate adversity and adapt to abiotic stress. Specifically, WRKY47, ERF96 and RAP2.6 have been found to be vital for plant response to Se stress in *Arabidopsis* [39,54,55]. The transcriptome data revealed 33 DEGs belonging to 15 TF families, including zf-C2H2, zf-C5HC2, zf-GRF, zf-NF-X1, HLH, GATA, bZIP_2, TEA, Fork_head, Homeobox, HSF_DNA-bind, CBFB_NFYA, SART-1, SRF-TF, and SGT1 (Supplementary Table S11). This significant enrichment of TFs highlighted their essential role in the evolution of selenite resistance.

Among these 15 TFs, zf-C2H2, zf-C5HC2, GATA, bZIP_2, TEA, Fork_head, Homeobox, and HSF_DNA-bind showed higher expressions in *G. lucidum* JNUSE-200 compared to *G. lucidum* CGMCC 5.26 grown without selenite; only the expression of one, HLH, was lower in *G. lucidum* JNUSE-200 than G. lucidum CGMCC 5.26. For both *G. lucidum* JNUSE-200 and *G. lucidum* CGMCC 5.26 grown with selenite, the expression levels of 25 TF genes varied remarkably: unigenes homologous to zf-C2H2, zf-C5HC2, GATA, TEA, Fork_head, and homeobox exhibited higher expression levels in *G. lucidum* JNUSE-200. These TFs might regulate the expression of key genes and contribute to selenite tolerance.

### 3.7. Correlation Coefficients: Transcription Factor–Key Gene–Se Bioaccmulation Capacity

Based on the aforementioned analysis, we chose the inorganic P transporter, sulfite efflux pump, CysJ, CysI, CysK, high-affinity methionine permease, fungal hydrophobin, putative chitin synthase 5, and APX as key target genes, and co-expression correlation coefficients with 33 TFs were calculated. Subsequently, five TFs that might regulate the five target genes were screened (Supplementary Table S12).

**Figure 9:**
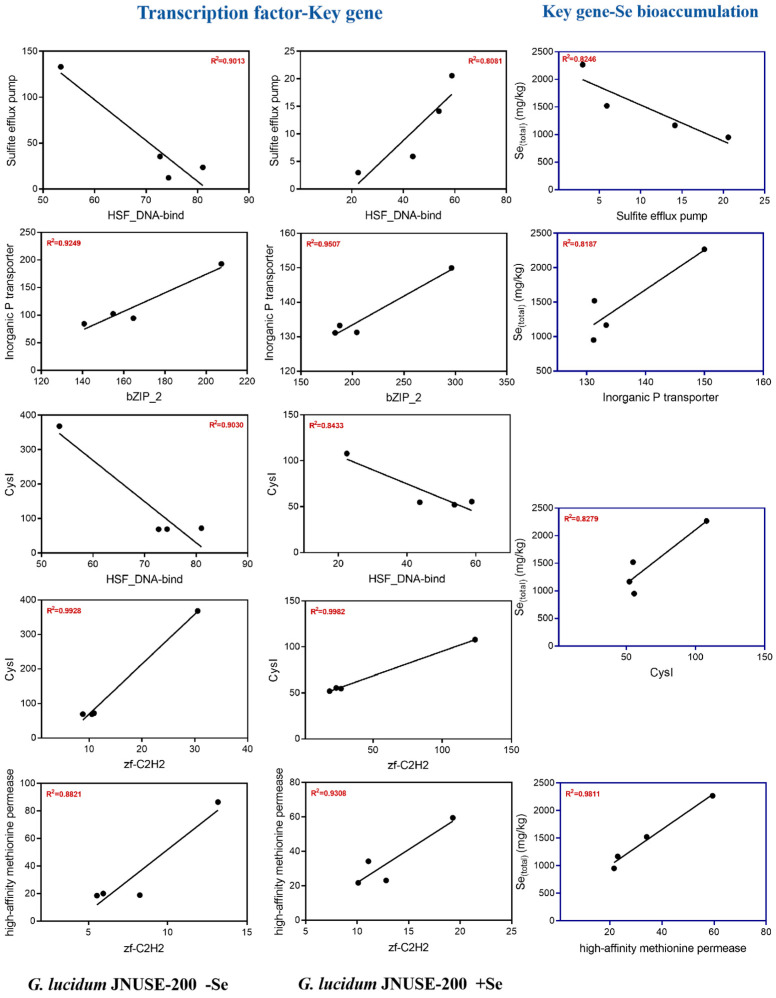
Correlation analysis of transcription factor–key gene–Se bioaccumulation capacity.

Further correlation coefficient analysis was conducted based on key genes and Se accumulation ability. The inorganic P transporter (GL23411-G), sulfite efflux pump (GL19873-G), CysI (GL18754-G), and high-affinity methionine permease (GL20912-G) were positively correlated with the Se accumulation capacity of *G. lucidum* JNUSE-200 mycelia, with R square values of 0.8246, 0.8287, 0.8279, and 0.9811, respectively (Figure 9). These four crucial genes may play a regulatory role in selenite tolerance in *G. lucidum* JNUSE-200.

### 3.8. RT-qPCR Verification of Differential Expression Genes

The expression patterns of the DEGs in our transcriptome data were validated by comparing the relative expression values of four key genes in *G. lucidum* JNUSE-200 with those in *G. lucidum* CGMCC 5.26 under two culture conditions. Consistent gene expression was observed for the sulfite efflux pump (GL19873-G), inorganic phosphate transporter (GL23411-G), high-affinity methionine permease (GL20912-G), and CysI (GL18754-G) between RT-qPCR and RNA-Seq data under both culture conditions, as shown in Supplementary Figure S4. These findings further support the reliability of our RNA-Seq data.

### 3.9. Mechanism of Enhanced Selenite Tolerance in G. lucidum JNUSE-200

Selenite stress has a detrimental impact on the growth and productivity of plants and microorganisms. It is widely accepted that, at equivalent concentrations, Se can stimulate the growth of Se-accumulating species while exerting toxic effects on non-Se-accumulating species. The primary mechanisms underlying Se toxicity in plants have been extensively investigated and summarized, including the competition between Se and S in both primary and secondary metabolism, incorporation of SeCys and SeMet into functional proteins, as well as metabolic disturbances and disruption of cell structure induced by oxidative stress [49]. Nature harbors several plant species with tolerance to selenite, such as *A. thaliana*, *S. pinnata*, and *Cardamine violifolia* [16,50,56]. Detailed mechanisms conferring selenite tolerance in well-studied plants are summarized in Table 1.

**Figure 10:**
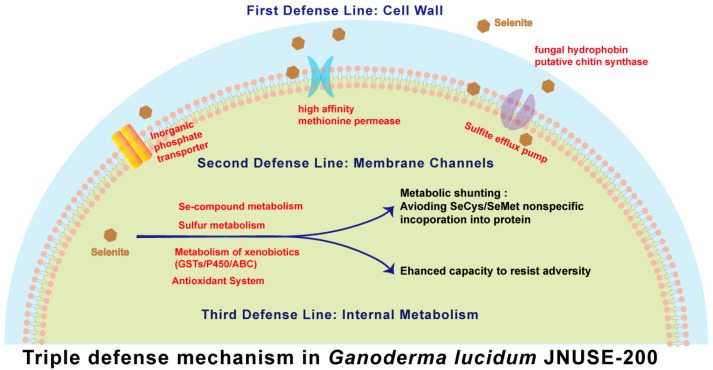
Molecular elucidation of selenite tolerance and hyperaccumulation in *G. lucidum*.

In this study, *G. lucidum* JNUSE-200 exhibited a triple defense mechanism during adaptive evolution (Figure 10). The first line of defense involves dispersed mycelial morphology and thickened cell walls. The second line of defense consists of three crucial cell membrane channels regulating selenite absorption. Lastly, the internal mechanism difference encompasses metabolic pathway shunting along with synergistically enhanced antioxidant mechanisms. Understanding the mechanisms responsible for selenite tolerance and identifying heritable properties are pivotal for enhancing the viability of improved sources for Se bioremediation purposes—a matter greatly significant for developing effective environmental treatments against Se pollution.

### 3.10. Implication

The concentrations of selenium in soils exhibit wide variations globally, ranging from 0 to 100 μg/g, with certain regions even reaching levels as high as 1200 μg/g. Excessive intake of Se can have detrimental effects on human health, emphasizing the significance of mitigating Se pollution in recent decades. Hyperaccumulating plants such as *Allium cepa*, *B. juncea*, and *S. pinnata* have been extensively utilized for environmental remediation of Se [2]. Mushrooms also possess remarkable capacity to accumulate Se due to their tolerance capabilities and serve as significant bioremediation resources for addressing this concern. In this study, *G. lucidum* JNUSE-200 was obtained through adaptive evolution from *G. lucidum* CGMCC 5.26, exhibiting enhanced selenite tolerance. Physiological and molecular responses to high levels of selenite were compared between the two strains of *G. lucidum* to elucidate the mechanism underlying their tolerance. These findings contribute to a better understanding of fungal Se tolerance and accumulation, provide theoretical guidance for breeding varieties that accumulate Se, and offer potential applications in bioremediation efforts targeting Se contamination.

## 4. Conclusions

Our study represents the first comprehensive analysis of mycelial physiological and transcriptomic changes in two strains of *G. lucidum* with varying capacities for selenite tolerance. *G. lucidum* JNUSE-200 has developed a triple defense mechanism to protect itself against high levels of selenite during adaptive evolution. The dispersion of mycelia and thickening of cell walls contribute to enhanced selenite tolerance in *G. lucidum* JNUSE-200. Furthermore, we have identified three membrane channel genes, including an Inorganic P transporter, a sulfite efflux pump, and high-affinity methionine permease genes, which play a crucial role in reducing selenite uptake and improving tolerance. Differences in internal mechanisms, particularly related to Se metabolism and sulfur metabolism, significantly influence the diversion of selenite to prevent non-specific incorporation of SeCys and SeMet into protein synthesis as a means to mitigate Se toxicity. This study provides novel insights into the molecular responses associated with selenite tolerance and accumulation in fungi, offering theoretical guidance for breeding and cultivating varieties that accumulate Se while also facilitating the application of fungal-mediated transformations for efficient, eco-friendly, and cost-effective methods for the remediation of Se-contaminated soil, sediments, and wastewater.

## Figures and Tables

**Table 1:** Summary of Se tolerance and accumulation mechanism.

Plant	Target Gene	Mechanism	Reference
*Arabidopsis*	Transporter gene	Sultr1;2 mutation conferred Se tolerance	[57]
	Metabolism related gene	*O*-acetylserine-(thiol) lyase A and B degrade L-Cys and L-SeCys and confer improved resistance	[49]
		APR2 mutant exhibited decreased selenate tolerance through disruption glutathione biosynthesis	[58]
		Increased AtCpNifS expression enhanced selenite tolerance	[50]
		Loss of function of terpenoid synthase (TPS22) enhanced Se tolerance	[38]
	Antioxidant gene	Loss of function of ascorbate peroxidase (APX1) enhanced selenite tolerance	[59]
	Signaling pathway gene	Ethylene and jasmonic acid signaling regulated selenite resistance	[60]
	Transcription factor gene	Gain- and loss-of-function mutations in WRKY47 enhanced the sensitivity to Se stress	[54]
		Increased ERF96 expression enhanced Se tolerance	[55]
		Increased RAP2.6 expression improved resistance to Se	[39]
	Metabolites	Glutathione	[61]
*Stanleya pinnata*	Metabolism related gene	The high ATP sulfurylase 2 activity in the cytosol and concomitant reduced ATPS activity in the plastids diverted Se fluxes	[56]
	Metabolism related gene	Up-regulated JA, SA and ethylene-mediated defense systems, elevated expression of genes in sulphate/selenate uptake and assimilation or in antioxidant activity	[13]
	Antioxidant gene	Increased antioxidants and up-regulated sulfur assimilation	[12]
*Cardamine violifolia*	Metabolism related gene	The downregulation of cysteine-rich kinases and calcium proteins enhanced Se tolerance	[62]
	Metabolites	Flavonoid, glutathione, and lignin	[15]
		Selenolanthionine	[16]
*Arachis hypogaea* L.	Antioxidant gene	Activating the antioxidant enzymes and mediating the ascorbate-glutathione cycle	[63]
*Nicotiana tabacum*	Transporter gene	Increased Pi transporter OsPT8 gene expression improved Se content	[37]
*Rice* ZH11	Metabolism related gene	Increased selenocysteine lyase and selenocysteine methyltransferase gene expressions enhanced selenate and selenite tolerance	[64]

## Data Availability

Data have been deposited in the China National Microbiology Data Center (NMDC) with accession number NMDC10018415 (https://nmdc.cn/resource/genomics/project/detail/NMDC10018415 (accessed on 15 May 2023)).

## Supplementary Materials

The following supporting information can be downloaded at https://www.mdpi.com/article/10.3390/jof10060415/s1: Figure S1: The nonlinear curve fit of growth curve related to Se in both *G.lucidum* CGMCC5.26 and *G.lucidum* JNUSE-200; Figure S2: DEGs of the Se-compound metabolism pathway in C-200-5d vs. H-200-5d; Figure S3: DEGs of the S metabolism pathway in C-200-5d vs. H-200-5d; Figure S4: RT-qPCR of 4 transcripts. The data represent the mean ± SD values of three replicates (*t*-test). The fold change is denoted as the ratio of the transcription level in the *G. lucidum* JNUSE-200 to that in *G. lucidum* CGMCC 5.26 with the same culture condition. Table S1: Primers used in this study; Table S2: Overview of raw and clean reads in two *G. lucidum* exposed to 0 or 200 mg/kg selenite; Table S3: DEGs related to mycelia cell wall; Table S4: DEGs related to membrane channels in *G. lucidum* CGMCC 5.26 and *G. lucidum* JNUSE-200; Table S5: DEGs in Se compound metabolism pathway; Table S6: DEGs in sulfur metabolism pathway; Table S7: DEGs of CYPs in *G. lucidum*; Table S8: DEGs of GSTs in *G. lucidum*; Table S9: DEGs of ABC transporters in *G. lucidum*; Table S10: Differently expressed antioxidant related genes; Table S11: Differently expressed transcription factors (TFs); Table S12: The co-expression correlation coefficients of target genes and transcription factors.
