# Supplementary material for: The Performance and Evolutionary Mechanism of Ganoderma lucidum in Enhancing Selenite Tolerance and Bioaccumulation
Source: J Fungi (Basel). 2024 Jun 8;10(6):415. doi: 10.3390/jof10060415 (PMC11205109; doi:10.3390/jof10060415)
Supplement: Supplementary file 1 [file jof-10-00415-s001.zip › Supplementary Figures.pdf]

The Performance and Evolutionary Mechanism of *Ganoderma lucidum* in Enhancing Selenite Tolerance and Bioaccumulation

Mengmeng Xu [1,2,3], Qi Meng [2,3], Song Zhu [4], Ruipeng Yu [4], Lei Chen [2,3], Guiyang Shi [2,3], Ka-Hing Wong [5], Daming Fan [1,4] and Zhongyang Ding [2,3,*]

1 School of Food Science and Technology, Jiangnan University, Wuxi 214122, China; xmm900801@163.com (M.X.); fandm@jiangnan.edu.cn (D.F.)

2 National Engineering Research Center for Cereal Fermentation and Food Biomanufacturing,
Jiangnan University, Wuxi 214122, China; mq15006177263@163.com (Q.M.); leichen@jiangnan.edu.cn (L.C.); gyshi@jiangnan.edu.cn (G.S.)

3 Jiangsu Provincial Research Center for Bioactive Product Processing Technology, Jiangnan University,
Wuxi 214122, China

4 State Key Laboratory of Food Science and Resources, Jiangnan University, Wuxi 214122, China;
zhusong@jiangnan.edu.cn (S.Z.); yuruipeng@jiangnan.edu.cn (R.Y.)

5 Research Institute for Future Food, Department of Food Science and Nutrition, The Hong Kong Polytechnic University, Hong Kong 999077, China; kahing.wong@polyu.edu.hk

* Correspondence: bioding@163.com or zyding@jiangnan.edu.cn ; Tel./Fax: +86-510-85918221

Supplementary Figures

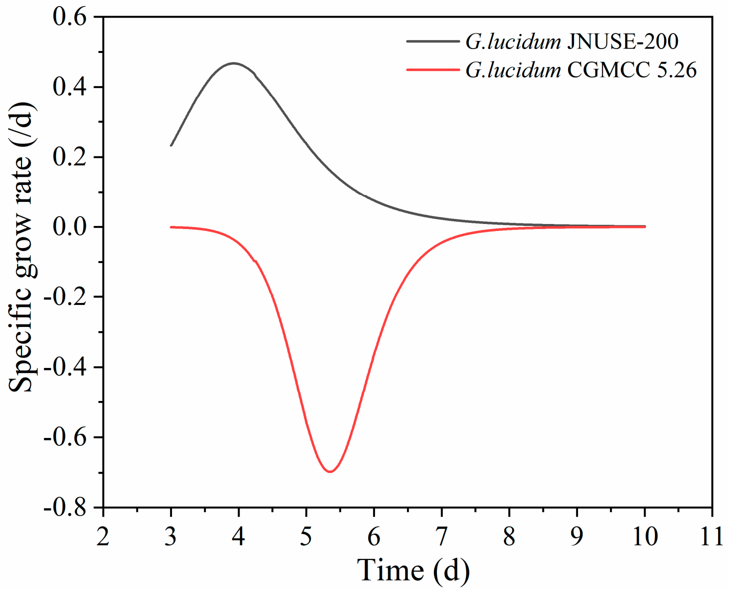

Figure. S1 The nonlinear curve fit of growth curve related to Se in both *G.lucidum* CGMCC5.26 and *G.lucidum* JNUSE-200

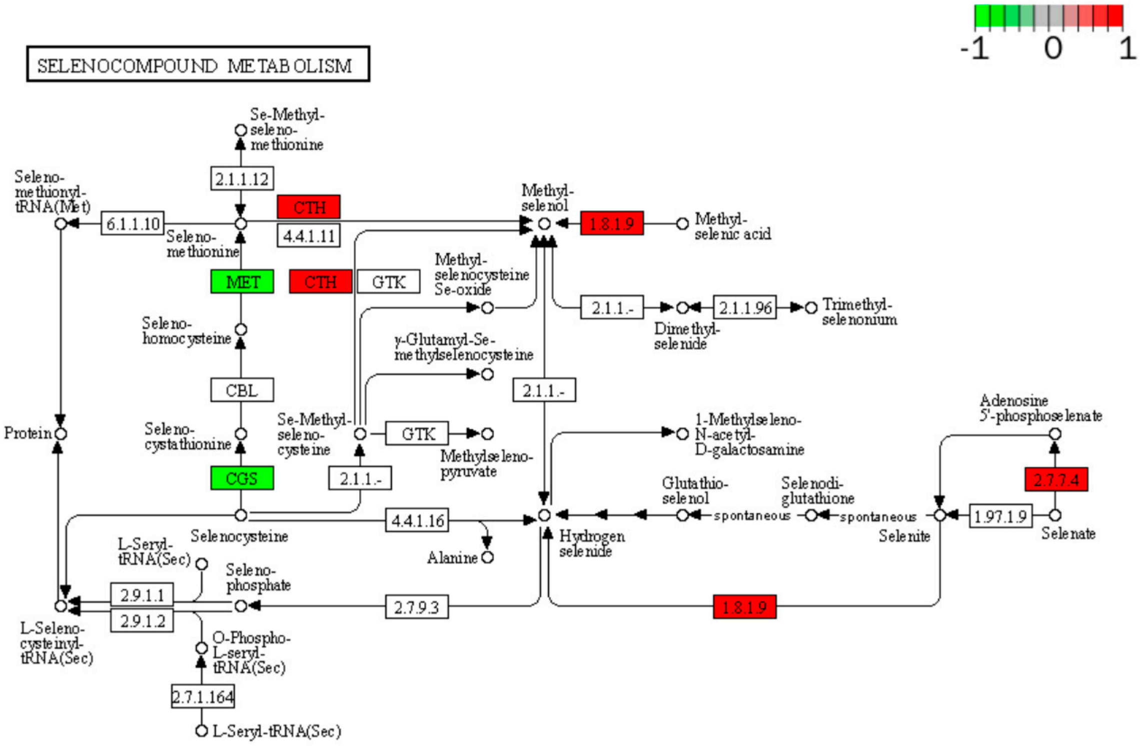

Figure. S2 DEGs of the Se-compound metabolism pathway in C-200-5d *vs* H-200-5d

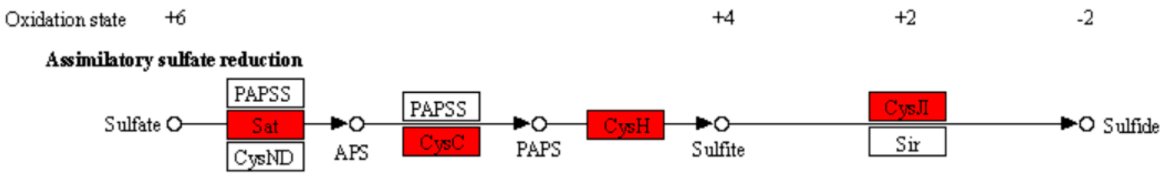

Figure. S3 DEGs of the S metabolism pathway in C-200-5d *vs* H-200-5d

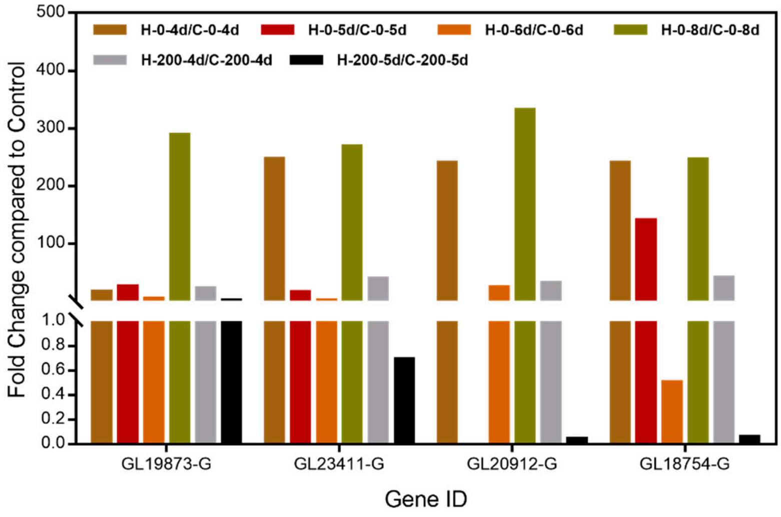

Figure. S4 RT-qPCR of 4 transcripts. The data represent the mean ± SD values of three replicates (*t*-test). The fold change is denoted as the ratio of the transcription level in the *G. lucidum* JNUSE-200 to that in *G. lucidum* CGMCC 5.26 with same culture condition.

Note: C-0: *G. lucidum* CGMCC 5.26 cultured without selenite; **C-200**: *G. lucidum* CGMCC 5.26 cultured without selenite; **H-0**: *G. lucidum* JNUSE-200 cultured without selenite; **H-200**: *G. lucidum* JNUSE-200 cultured without selenite.
